# Nucleoredoxin Gene *TaNRX1* Positively Regulates Drought Tolerance in Transgenic Wheat (*Triticum aestivum* L.)

**DOI:** 10.3389/fpls.2021.756338

**Published:** 2021-11-11

**Authors:** Yunrui Zhang, Jianfei Zhou, Fan Wei, Tianqi Song, Yang Yu, Ming Yu, Qiru Fan, Yanning Yang, Gang Xue, Xiaoke Zhang

**Affiliations:** ^1^College of Agronomy, Northwest A&F University, Xianyang, China; ^2^College of Tobacco, Henan Agricultural University, Zhengzhou, China

**Keywords:** wheat, nucleoredoxin, *TaNRX1*, drought, RNA-seq

## Abstract

Drought is the main abiotic stress factor limiting the growth and yield of wheat (*Triticum aestivum* L.). Therefore, improving wheat tolerance to drought stress is essential for maintaining yield. Previous studies have reported on the important role of *TaNRX1* in conferring drought stress tolerance. Therefore, to elucidate the regulation mechanism by which *TaNRX1* confers drought resistance in wheat, we generated *TaNRX1* overexpression (OE) and RNA interference (RNAi) wheat lines. The results showed that the tolerance of the OE lines to drought stress were significantly enhanced. The survival rate, leaf chlorophyll, proline, soluble sugar content, and activities of the antioxidant enzymes (catalase, superoxide dismutase, and peroxidase) of the OE lines were higher than those of the wild type (WT); however, the relative electrical conductivity and malondialdehyde, hydrogen peroxide, and superoxide anion levels of the OE lines were lower than those of the WT; the RNAi lines showed the opposite results. RNA-seq results showed that the common differentially expressed genes of *TaNRX1* OE and RNAi lines, before and after drought stress, were mainly distributed in the plant–pathogen interaction, plant hormone signal transduction, phenylpropane biosynthesis, starch and sucrose metabolism, and carbon metabolism pathways and were related to the transcription factors, including WRKY, MYB, and bHLH families. This study suggests that *TaNRX1* positively regulates drought stress tolerance in wheat.

## Introduction

Thioredoxin (TRX) is a class of small proteins with a molecular weight of approximately 12 kDa widely found in animals, plants, fungi, and microorganisms (Meyer et al., 2012). TRX contains a conserved active site domain, WCG/PPC, and two cysteine (Cys) residues in the active center that participate in the redox reaction through the reversible conversion of thiol–disulfide to maintain the stability of the cell environment (Powis and Montfort, 2001; Marx et al., 2003). TRX plays an irreplaceable role in maintaining the balance of the intracellular redox state and regulating the spatial structure and activity of the proteins. It is also involved in regulating plant growth and development and the response process in resisting drought, high temperature, and other adverse stresses (Marx et al., 2003). TRX participates in a series of physiological and biochemical processes in cells by reducing disulfide bonds in the target proteins (Wong et al., 2004; Joudrier et al., 2005; Traverso et al., 2007; Schurman and Buchanan, 2008). Nucleoredoxin (NRX) is a member of the TRX family, and its molecular weight is considered to be larger than that of TRX.

Nucleoredoxin in plants was first isolated from maize (*Zea mays* L.) by Laughner et al. (1998). Based on the number of TRX-like domains and its amino acid sequence, the plant NRX can be classified into three types. Type I NRX contains three TRX-like domains; the first and third domains contain typical WCG/PPC redox active sites, and the second domain is an atypical TRX-like domain. Type II NRX contains two TRX-like domains and the atypical TRX active sites (WYP/AK/PC and W/R/HCL/A/V/RPC/G). Type III NRX contains two TRX-like domains, including the highly conserved WCRPC redox active site and the typical TRX active site WCPPC/F/S (Marchal et al., 2014). Laughner et al. (1998) found that the maize NRX consists of three TRX-like domains, of which the first and third domains contain the active site WCPPC of the typical TRX-like domain, and the third domain exhibits a disulfide bond reduction ability *in vitro*. Li et al. (2016) also found that the first and third domains of the cotton (*Gossypium barbadense* L.) GbNRX1 contain the active site WCG/CPC, which is a typical TRX-like domain; GbNRX1 also has an obvious disulfide bond reduction activity. Both *Arabidopsis thaliana* NRX type I (AtNRX1) and type II (AtNRX2) exhibit disulfide bond reduction abilities *in vitro* (Marchal et al., 2014). Cheng (2021) showed that the three domains of wheat (*Triticum aestivum* L.) TaNRX1 exhibit insulin disulfide bond reduction activity *in vitro*; among these domains, the third domain has the strongest reduction activity and the second domain has the weakest activity.

Funato et al. (2013) found that NRX directly interacts with phosphofructokinase 1 (PFK1) to regulate the activity of PFK1, thereby maintaining the balance between glycolysis and pentose phosphate pathways. Zhao (2016) showed that AtNRX1 is involved in the regulation of reactive oxygen species (ROS) levels in *Arabidopsis* seedlings. Kneeshaw et al. (2017) showed that NRX1 targets enzymes in the hydrogen peroxide (H_2_O_2_) clearance pathway, including catalase (CAT); an NRX1 mutant showed reduced CAT activity and was extremely sensitive to oxidative stress. NRX1 regulates CAT activity, enhances the detoxification ability of CAT, and protects antioxidant enzymes from oxidative stress induced by ROS, thereby protecting plant cells from oxidative stress (Kneeshaw et al., 2017). Evidently, NRX plays an important role in plants, and an understanding of NRX and its target proteins will help elucidate the molecular mechanism of NRX in plant resistance to adverse stresses.

Wheat is one of the most important food crops worldwide. Wheat production is often adversely affected by biotic and abiotic stresses (Tuan et al., 2019), among which drought is the main abiotic stress factor limiting global wheat production (Valluru et al., 2016). It severely affects the growth and development of wheat plants by causing various physiological and biochemical damage, resulting in serious wheat yield declines (Dey et al., 2016; Zhou et al., 2016; Ma J. et al., 2017; Saradadevi et al., 2017; Yadav et al., 2019). Therefore, a comprehensive understanding of the molecular mechanisms of drought resistance in wheat is important for increasing wheat yield and ensuring food security.

Previously, in our laboratory, Zhang et al. (2014) cloned a *TaNRX1* gene in common wheat and reported that *TaNRX1* is associated with drought resistance; however, the molecular mechanisms underlying drought resistance in wheat require further elucidation. In this study, *TaNRX1* overexpression (OE) and RNA interference (RNAi) transgenic wheat plants were subjected to drought stress treatment to elucidate the drought resistance mechanism of *TaNRX1*. This study identified an excellent candidate gene TaNRX1 for the production of drought-resistance wheat plants, selection and promotion of drought-resistant wheat varieties, and improvement in wheat yield and food security.

## Materials and Methods

### Plant Materials

The cDNA sequence of *TaNRX1* (NCBI ID: KC890769) was cloned from Jinmai 47, a drought-tolerant common wheat variety. The wheat variety JW1 and expression vector pLGY-02 were provided by Professor Genying Li (Crop Research Institute, Shandong Academy of Agricultural Sciences, China). The vector pLGY-02 used has been presented in Liang et al. (2020).

### Vector Construction and Generation of Transgenic Wheat

To generate transgenic plants overexpressing *TaNRX1*, the full-length coding sequence (CDS) of *TaNRX1* was cloned into the *Kpn*I-HF and *Spe*I-HF (Supplementary Table 1) (New England Biolabs, Beijing, China) sites of pLGY-02, a plant transformation vector driven by the maize ubiquitin promoter. The RNAi vector was constructed to silence *TaNRX1* in wheat, and two 223-bp truncated fragments (forward and reverse fragments) of *TaNRX1* CDS (Δ*TaNRX1*) were amplified and inserted into the pLGY-02 vector to form a hairpin structure (Δ*TaNRX1*-intron-Δ*TaNRX1*). The truncated fragments contained *Kpn*I and *Sac*I restriction sites. The constructed vector was then transformed into wheat (JW1) immature embryos via *Agrobacterium tumefaciens*-mediated transformation (Cheng et al., 1997).

### Screening of Homogeneous Transgenic Wheat Lines

In this experiment, specific primers HygF1 and HygR1 (Supplementary Table 1) of the transgenic vector hygromycin resistance gene were used to detect different lines of transgenic wheat in the T_1_ generation and to screen the seeds of the transgenic wheat with a resistance: sensitivity ratio of 3:1 to identify single-copy transgenic lines. Genomic DNA was isolated from transgenic wheat leaves using the cetyltrimethylammonium bromide method (Porebski et al., 1997). The DNA was then used for polymerase chain reaction (PCR) assays, and PCR-amplified products were separated by 1.0% agarose gel electrophoresis. The T_2_ generation of the transgenic wheat was detected by PCR, and the T_3_ generation was detected by PCR and RT-qPCR.

### Reverse Transcription Quantitative PCR

Total RNA was extracted from the leaves of the transgenic wheat using TRNzol Universal Reagent DP424 (TianGen Biotech, Beijing, China). cDNA was synthesized using EasyScript^®^ One-Step gDNA Removal and cDNA Synthesis SuperMix AE311-02 (TransGen Biotech, Beijing, China). To determine the transgenic wheat lines and drought stress periods for RNA-seq, RT-qPCR was performed using the TB Green Premix Ex Taq (Takara, Beijing, China). Each reaction was performed in triplicate. In addition, RT-qPCR was used to determine the relative expression levels of *TaNRX1* in WT and transgenic wheat lines (*TaNRX1-OE-1, TaNRX1-OE-3*, and *TaNRX1-OE-6* and *TaNRX1-RNAi-6, TaNRX1-RNAi-8*, and *TaNRX1-RNAi-9*) during different drought stress periods. All primers used in the experiments are listed in Supplementary Table 1. The actin gene was used as an endogenous control for template cDNA normalization, and the 2^–ΔΔ^ *^*C*^*^*T*^ method (Livak and Schmittgen, 2001) was used to calculate the relative expression levels.

### Drought Stress Treatment

To determine the effect of drought stress on roots during the seedling stage, the germinated seeds of WT, OE, and RNAi wheat lines were cultivated with 1/2 Hoagland nutrient solution and 1/2 Hoagland nutrient solution with 20% (M/V) polyethylene glycol (PEG6000). Plants were cultivated in a light incubator at 20–25°C, with a 16-h-light/8-h-dark photoperiod, with humidity being approximately 70% and light intensity being 18,000 Lux. After 10 days of seed germination, morphological and physiological parameters of plant roots were measured once the final age of seedlings was 14 days.

To analyze the drought tolerance of wheat plants at the seedling stage, WT and *TaNRX1* transgenic wheat seeds were germinated in petri dishes and transplanted into pots containing the same soil volumes (10 plants per pot) in a greenhouse at 15–20°C, with a 16-h-light/8-h-dark photoperiod for 4 weeks. Control plants were watered normally, and drought stress in plants was induced by withholding watering for 10 days when the relative water content of the soil was approximately 60%; the physiological indexes of the WT and transgenic wheat lines under control and drought stress conditions were measured. For 4-week-old WT, OE, and RNAi wheat plants, watering was withheld for 14 days when the soil relative water content was below 50%; rewatering was resumed after 3 days, and the phenotype and survival rates were recorded.

To analyze the drought resistance of the wheat at the heading stage, WT and *TaNRX1* transgenic wheat lines were grown in pots with the same soil volumes (six plants per pot) in a greenhouse at 15–20°C, with a 16-h-light/8-h-dark photoperiod. The water supply of 45-day-old plants was stopped when the tip of the main spike emerged from the flag leaf sheath (Zhang et al., 2017). The soil relative water content (RWC) of the drought-stressed plants was maintained at 35–40%, and the control plants were watered normally. At 21 days after drought treatment, the phenotype was observed and chlorophyll content and photosynthesis-related indicators were measured. All drought stress treated plants were homogenous lines of the transgenic wheat T_3_ generation.

### Measurement of Drought-Relevant Physiological Indicators

Wild type, OE, and RNAi wheat plants were used to measure the water loss rate according to Srivastava’s method (Srivastava et al., 2017). RWC was measured according to the method described by Chen et al. (2016). The content of proline and soluble sugar were measured according to the method described by Wang N. et al. (2017). Malondialdehyde (MDA) content was measured using the thiobarbituric acid method (Zhang et al., 2020). Relative electrical conductivity (REC) was determined according to the method described by Ma Y. et al. (2017).

Hydrogen peroxide (H_2_O_2_) and superoxide anions (O_2_^–^) were detected using 3,3-diamino-benzidinestaining (DAB) and nitroblue tetrazolium (NBT) staining, respectively. For DAB staining, the wheat control and drought stress leaves were immersed in DAB staining solution (0.4 mg/mL) for 4 h and then decolorized in an ethanol:acetic acid:glycerol solution at a ratio of 3:1:1 (V/V/V). For NBT staining, the wheat leaves were immersed in NBT staining solution (0.1 mg/mL) for 6 h and then decolorized according to the NBT staining method (Wang N. et al., 2017). Images were obtained using an Olympus stereo microscope (Olympus, Tokyo, Japan). The H_2_O_2_ content was measured using a H_2_O_2_ test box (Suzhou Keming Biotechnology Co., Ltd., Suzhou, China). The O_2_^–^ level was measured according to the method described by Wang K. et al. (2017). The superoxide dismutase (SOD), catalase (CAT), and peroxidase (POD) contents were measured using an SOD, CAT, and POD test box (Suzhou Keming Biotechnology Co., Ltd., Suzhou, China). The photosynthesis-related indicators at the heading stage were measured using a portable photosynthetic system (LI-6400XT, United States). In this study, at least three replicates were performed for each experiment.

### RNA Sequencing

T_3_ homozygous lines of transgenic wheat and the WT were used for RNA-seq. Wheat seedlings were cultured in the same pot with 1/2 Hoagland nutrient solution for 2 weeks and then transferred into fresh 1/2 Hoagland nutrient solution containing 20% PEG6000. Leaves were collected at 0 and 6 h following drought treatment, and three biological replicates were used for RNA-seq.

RNA extraction and RNA-seq analysis were performed by Biomarker Biotech Company (Beijing, China). The RNA integrity number, with ≥7 samples, was selected for subsequent analysis. RNA-seq was performed using Illumina Novaseq 6000 system with paired-end sequencing and 150 bp sequencing reads. The Pearson correlation coefficient between each pair of biological replicates was calculated to confirm the reliability of the RNA-seq data. Gene expression levels were measured based on fragments per kilobase of transcript per million fragments mapped values. For significant differentially expressed genes (DEGs), log_2_[fold change] ≥ 2 and a false discovery rate < 0.05 were used as the screening criteria. Gene Ontology (GO) enrichment analysis was used to determine the main biological functions of DEGs, and Kyoto Encyclopedia of Genes and Genomes (KEGG) enrichment analysis was used to determine the main physiological and biochemical metabolic pathways of DEGs. All RNA-seq data were analyzed using the Biomark Cloud Platform (BMKCloud^1^). To verify the reliability of the RNA-seq data, we randomly selected seven DEGs to determine their relative expression levels by RT-qPCR. RT-qPCR was performed as described in Section “Reverse Transcription Quantitative PCR.”

### Statistical Analysis

All experiments were independently repeated at least three times to obtain reliable data for statistical analysis. Statistical analysis was performed using SPSS 20.0 (SPSS, Chicago, IL, United States). The *parametric tests* one-way analysis of variance was used for data analysis, and the least significant difference *post hoc* was used to determine significant differences between means (*, statistically significant at *P* < 0.05; **, statistically significant at *P* < 0.01).

## Results

### Generation and Identification of *TaNRX1* Transgenic Wheat Lines

To study the drought resistance and molecular mechanism of *TaNRX1* in wheat, we used *TaNRX1* OE and RNAi-silenced wheat lines. A total of 140 embryos were infected; among these, after screening and regeneration, 16 OE and 15 RNAi plants were obtained.

**FIGURE 1:**
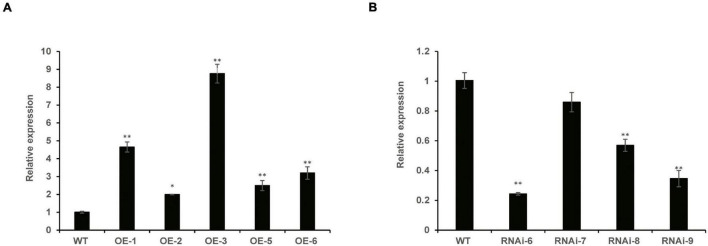
The relative expression of *TaNRX1* in transgenic wheat. **(A)** The relative expression of *TaNRX1* in overexpression lines (*n = 3*). **(B)** The relative expression of *TaNRX1* in RNA interference lines (*n = 3*). Data represent the mean ± SD. WT, wild type; OE-1-OE-6, *TaNRX1* overexpression T_3_ homogeneous lines; RNAi-6–RNAi-9, *TaNRX1* RNA interference T_3_ homogeneous lines. *P < 0.05, **P < 0.01 represent significant difference between the transgenic line and the WT, respectively.

To identify homozygous transgenic wheat lines, T_1_–T_3_ generations were confirmed by PCR. The vector pLGY-02 contained hygromycin resistance, and the 950 bp DNA segment was amplified in the transgenic wheat lines; however, no amplification was detected in WT plants (Supplementary Figure 1). RT-qPCR analysis demonstrated that the relative expression levels of *TaNRX1* in the three OE lines (*TaNRX1-OE-1, TaNRX1-OE-3*, and *TaNRX1-OE-6*) were significantly (*P* < 0.05) higher than those in the WT (Figure 1A). Similarly, the relative expression levels of *TaNRX1* in three RNAi lines (*TaNRX1-RNAi-6, TaNRX1-RNAi-8*, and *TaNRX1-RNAi-9*) decreased by approximately 76, 44, and 66%, respectively (Figure 1B). These results confirmed that *TaNRX1* was integrated and expressed in the wheat genome; the OE (*TaNRX1-OE-1, TaNRX1-OE-3*, and *TaNRX1-OE-6*) and RNAi (*TaNRX1-RNAi-6, TaNRX1-RNAi-8*, and *TaNRX1-RNAi-9*) lines were selected for subsequent experiments. To understand whether other TRX or NRX members were down-regulated by off-targeting, we compared expression levels of TRX and NRX members in WT and RNAi lines that were not subjected to drought stress (drought stress 0 h) in RNA-seq data. According to whether the protein has at least one Thioredoxin domain, a total of 109 TRX and NRX members were identified. Results showed that compared with expression levels of TRX and NRX members in WT, those of other TRX and NRX members in the RNAi lines were not down-regulated (Supplementary Table 2), indicating no off-targeting, and therefore the RNAi lines were used for subsequent experiments.

### Drought Resistance of *TaNRX1* Transgenic Wheat at the Seedling and Heading Stages

**FIGURE 2:**
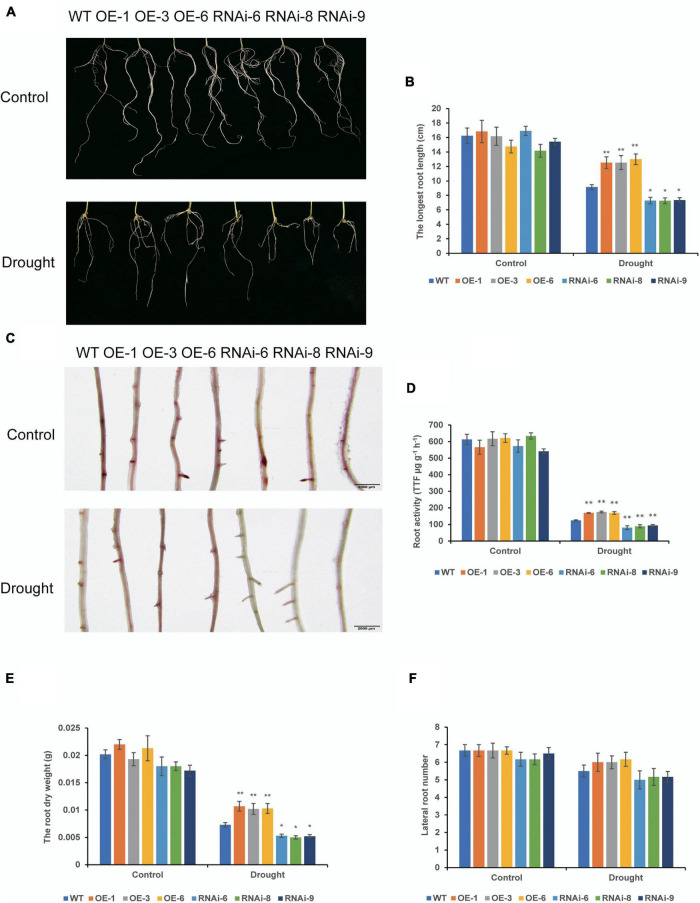
Effect of drought stress on the root system of WT and transgenic wheat at the seedling stage. **(A)** The roots phenotypes of WT and *TaNRX1* transgenic wheat were cultured in 1/2 Hoagland nutrient solution and 1/2 Hoagland nutrient solution with 20% (M/V) polyethylene glycol (PEG6000) for 10 days. **(B)** The longest root length (*n* = 6). **(C)** 2, 3, 5-triphenyltetrazolium chloride (TTC) staining of roots. Scale bar = 2 mm. **(D)** Root activity (*n* = 3). **(E)** Root dry weight (*n* = 6). **(F)** Lateral root numbers (*n* = 6). Data represent the mean ± SD. WT, wild type; OE-1–OE-6, *TaNRX1* overexpression T_3_ homogeneous lines; RNAi-6–RNAi-9, *TaNRX1* RNA interference T_3_ homogeneous lines. *P < 0.05, **P < 0.01 represent the significant difference between the transgenic line and the WT, respectively.

To examine drought tolerance of transgenic wheat at different growth stages, we determined the drought resistance function of *TaNRX1* by subjecting transgenic wheat to drought stress at the seedling and heading stages. Under control conditions, root morphology between WT and transgenic wheat at the seedling stage were not differed (Figure 2A). After 10 days of drought stress, the average longest root length of the OE lines [*TaNRX1-OE-1* (12.5 cm), *TaNRX1-OE-3* (12.5 cm), and *TaNRX1-OE-6* (13.0 cm)] was significantly (*P* < 0.01) longer than that of the WT (9.17 cm), whereas that of the RNAi lines was significantly (*P* < 0.05) shorter than that of the WT (Figure 2B). After drought stress, the 2, 3, 5-triphenyltetrazolium chloride staining of the OE lines was darker and the root activity was higher than those of the WT; RNAi lines were light and had low root activity (Figures 2C,D). The root dry weight of the OE lines was significantly (*P* < 0.01) greater than that of the WT (Figure 2E); there was no significant difference of lateral root numbers between the WT and transgenic wheat lines (Figure 2F). The results show that *TaNRX1* OE enhanced root growth and activity under drought stress.

**FIGURE 3:**
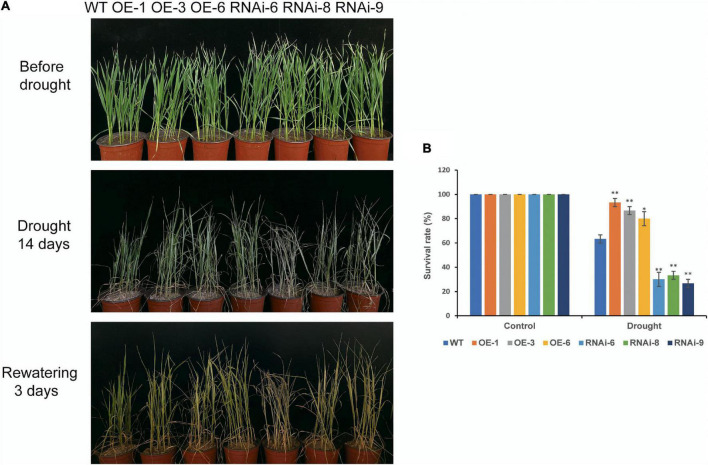
Phenotype and survival rate of WT and *TaNRX1* transgenic wheat under drought stress at the seedling stage. **(A)** WT and *TaNRX1* transgenic wheat phenotype at 4 weeks, after 14 days drought treatment, and 3 days rewatering. **(B)** Survival rate (*n* = 6). Data represent the mean ± SD. WT, wild type; OE-1–OE-6, *TaNRX1* overexpression T_3_ homogeneous lines; RNAi-6–RNAi-9, *TaNRX1* RNA interference T_3_ homogeneous lines. *P < 0.05, **P < 0.01 represent the significant difference between the transgenic line and the WT, respectively.

Before drought stress, there was no visibly discernible difference in phenotype between the WT and transgenic wheat plants at the seedling stage (Figure 3A). After 14 days of drought stress, both WT and transgenic wheat plants exhibited different wilting and yellowing. The difference between OE and WT was not obvious, but the wilting in RNAi lines were more severe than that in the WT (Figure 3A). The survival rates of the WT and transgenic wheat lines after rewatering for 3 days were significantly (*P* < 0.05) different. The survival rates of the OE lines [*TaNRX1-OE-1* (93.3%), *TaNRX1-OE-3* (86.7%), and *TaNRX1-OE-6* (80.0%)] were the highest, followed by the WT (63.3%); the survival rates of the RNAi lines [*TaNRX1-RNAi-6* (30.0%), *TaNRX1-RNAi-8* (33.3%), and *TaNRX1-RNAi-9* (26.7%)] were the lowest (Figure 3B). The relative expression of *TaNRX1* in WT, OE lines and RNAi lines under drought stress for 10 and 14 days were determined by qRT-PCR. After 10 and 14 days of drought stress, the expression levels of *TaNRX1* in OE lines were significantly (*P* < 0.01) higher than those of WT and RNAi lines, while the expression levels of *TaNRX1* in RNAi lines were still lower than WT (Supplementary Figure 2). These results indicate that *TaNRX1* OE enhanced wheat drought tolerance at the seedling stage, whereas RNAi of the *TaNRX1* gene significantly reduced drought resistance.

**FIGURE 4:**
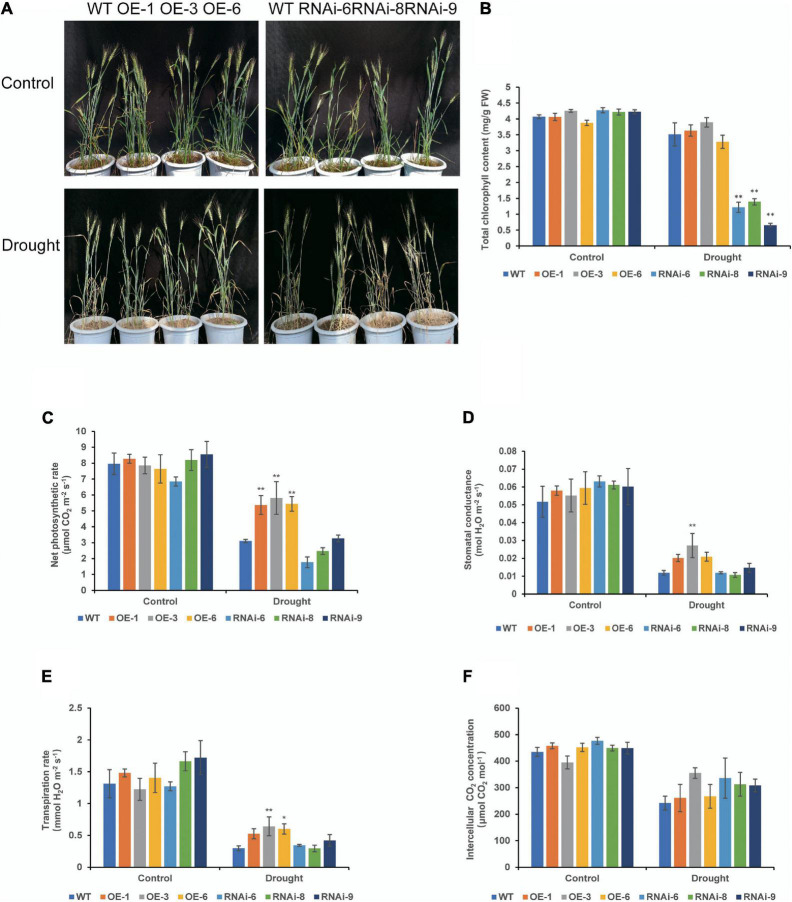
Phenotype and photosynthetic parameters of WT and *TaNRX1* transgenic wheat under drought stress at the heading stage. **(A)** Phenotype of 45-day-old WT and *TaNRX1* transgenic wheat in normal water supply and 21 days of drought stress (the soil relative water content was 35–40%). **(B)** Total chlorophyll content (*n* = 4). **(C)** Net photosynthetic rate (*n* = 6). **(D)** Stomatal conductance (*n* = 6). **(E)** Transpiration rate (*n* = 6). **(F)** Intercellular CO_2_ concentration (*n* = 6). Data represent the mean ± SD. WT, wild type; OE-1–OE-6, *TaNRX1* overexpression T_3_ homogeneous lines; RNAi-6–RNAi-9, *TaNRX1* RNA interference T_3_ homogeneous lines. *P < 0.05, **P < 0.01 represent the significant difference between the transgenic line and the WT, respectively.

There were no major growth defects in OE and RNAi compared to those in WT under normal conditions, OE lines were denser than WT, while RNAi lines were less dense than WT at the heading stage (Figure 4A). After drought stress treatment, leaves of both WT and transgenic wheat lines turned yellow and wilted. Leaf wilt of OE lines were less severe, while that of WT and RNAi lines were more severe (Figure 4A). Under normal conditions, there was no significant difference in the total chlorophyll content between the WT and transgenic wheat lines. After 21 days of drought stress, the total chlorophyll content of the RNAi lines was significantly (*P* < 0.01) lower than that of the WT and OE lines (Figure 4B). There was no distinct difference in the net photosynthetic rate (Pn) and other photosynthetic indicators between the WT and transgenic wheat flag leaves under control conditions. After drought stress controlled the soil RWC at 35–40% for 21 days, the Pn of the OE lines was significantly (*P* < 0.01) higher than that of the WT (Figure 4C). The stomatal conductance and transpiration rate were similar to those of Pn, while those of OE lines were higher than those of WT and RNAi lines. The OE-3 was significantly different (*P* < 0.01), and the rest of the lines were not significantly different (*P* > 0.05) (Figures 4D,E). There was no significant difference in intercellular CO_2_ concentration (Figure 4F). These results suggest that *TaNRX1* OE can improve the photosynthetic performance of wheat under drought stress.

### Effects of Drought Stress on the Water Retention of Transgenic Wheat

**FIGURE 5:**
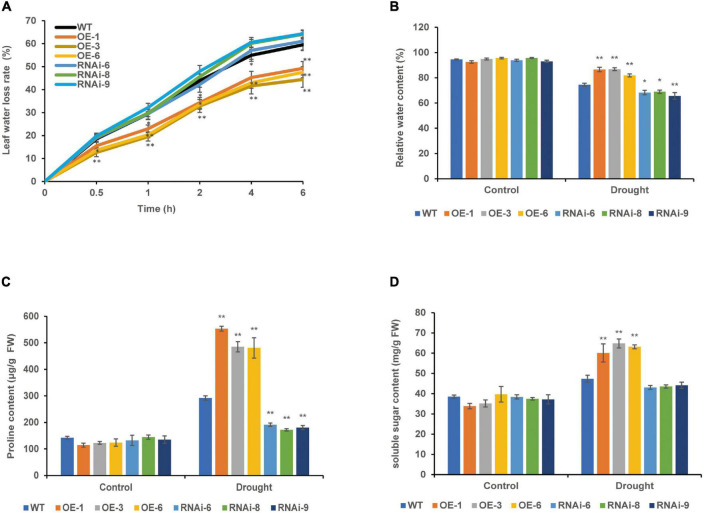
Effect of drought stress on the water status of WT and transgenic wheat. **(A)** Leaf water loss rate of 4 weeks WT and *TaNRX1* transgenic wheat (*n* = 15). **(B)** Relative water content of 4 weeks WT and *TaNRX1* transgenic wheat in normal water supply and 10 days of drought stress (*n* = 6). **(C)** Proline content (*n* = 4). **(D)** Soluble sugar content (*n* = 4). Data represent the mean ± SD. WT, wild type; OE-1–OE-6, *TaNRX1* overexpression T_3_ homogeneous lines; RNAi-6–RNAi-9, *TaNRX1* RNA interference T_3_ homogeneous lines. *P < 0.05, **P < 0.01 represent the significant difference between the transgenic line and the WT, respectively.

The leaf water loss rate of 4-week-old WT, OE, and RNAi wheat lines increased gradually with the prolonging of leaf detachment time; the leaf water loss rate of the OE lines was significantly (*P* < 0.05) lower than that of the WT for 0.5–6 h *in vitro*, the RNAi lines was higher than that of the WT, but the difference was not significant (*P* > 0.05) (Figure 5A). There was no significant difference in RWC between the WT and transgenic wheat lines under the control conditions. After drought stress, the RWC of both the WT and transgenic wheat showed a downward trend. The RWC of the OE lines was the highest and significantly (*P* < 0.01) increased by 14.23% compared with that of the WT, whereas the RWC of the RNAi lines was the lowest and significantly (*P* < 0.05) decreased by 9.16% compared with that of the WT (Figure 5B).

Under control conditions, the proline and soluble sugar contents were not significantly different between the WT and transgenic wheat lines. After 10 days of drought stress, the proline content of the OE lines [*TaNRX1-OE-1* (553.3 μg/g), *TaNRX1-OE-3* (485.0 μg/g), and *TaNRX1-OE-6* (480.9 μg/g)] was significantly (*P* < 0.05) higher than that of the WT (292.0 μg/g), whereas the proline content of the RNAi lines [*TaNRX1-RNAi-6* (191.2 μg/g), *TaNRX1-RNAi-8* (172.2 μg/g), and *TaNRX1-RNAi-9* (180.1 μg/g)] was significantly (*P* < 0.05) lower than that of the WT (291.7 μg/g) (Figure 5C). The soluble sugar in leaves also showed the same result under drought stress; the soluble sugar content of the OE lines [*TaNRX1-OE-1* (60.1 mg/g), *TaNRX1-OE-3* (64.8 mg/g), and *TaNRX1-OE-6* (63.2 mg/g)] was significantly (*P* < 0.05) higher than that of the WT (47.3 mg/g) (Figure 5D). Thus, under drought stress, *TaNRX1* OE in transgenic wheat was associated with the accumulation of osmotic adjustment substances, such as proline and soluble sugar, and enhances water retention ability, thereby reducing water loss after drought stress and enhancing the water retention capacity of wheat.

### Effects of Drought Stress on the Reactive Oxygen Species Levels of Transgenic Wheat

**FIGURE 6:**
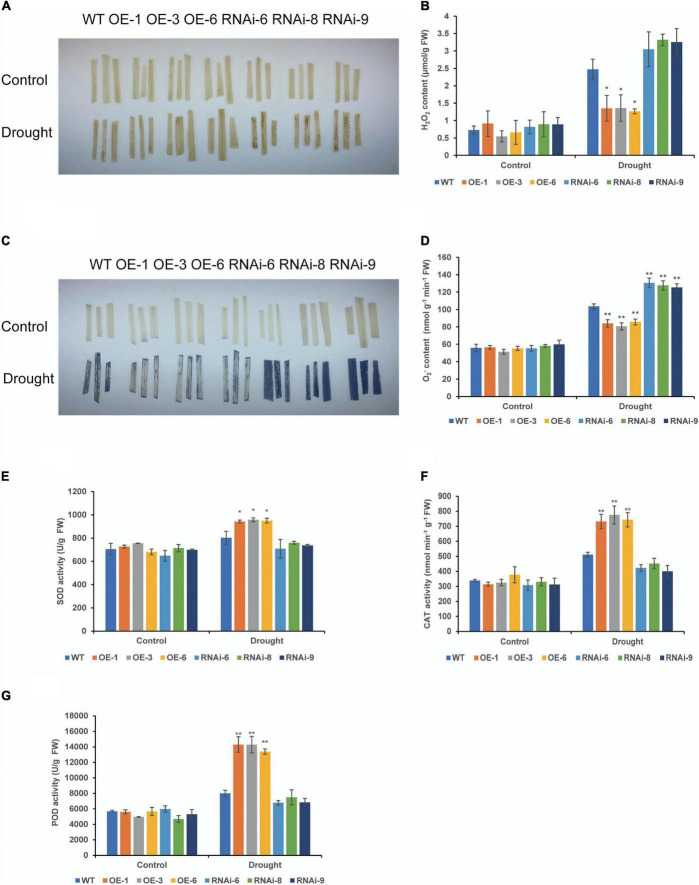
Effect of drought stress on the ROS of WT and transgenic wheat. **(A)** Histochemical staining was used to detect H_2_O_2_ by DAB staining with 4 weeks WT and *TaNRX1* transgenic wheat in normal water supply and 10 days of drought stress. **(B)** H_2_O_2_ content (*n* = 3). **(C)** Histochemical staining was used to detect O_2_^–^ by NBT staining. **(D)** O_2_^–^ content (*n* = 4). **(E)** Superoxide dismutase (SOD) content (*n* = 4). **(F)** Catalase (CAT) content (*n* = 3). **(G)** Peroxidase (POD) content (*n* = 4). Data represent the mean ± SD. WT, wild type; OE-1–OE-6, *TaNRX1* overexpression T_3_ homogeneous lines; RNAi-6–RNAi-9, *TaNRX1* RNA interference T_3_ homogeneous lines. *P < 0.05, **P < 0.01 represent the significant difference between the transgenic line and the WT, respectively.

To clarify the effect of *TaNRX1* OE or RNAi on wheat ROS levels, we measured H_2_O_2_, O_2_^–^, and antioxidant enzyme activity in WT, OE, and RNAi wheat. Under normal conditions, DAB and NBT staining results between the WT and transgenic wheat lines were not varied. After 10 days of drought stress, DAB staining of the RNAi lines was the darkest, followed by the WT, and that of the OE lines was the lightest (Figure 6A). The quantitative determination of H_2_O_2_ also showed that the accumulation of H_2_O_2_ in the leaves of the OE lines [*TaNRX1-OE-1* (1.3 μmol/g), *TaNRX1-OE-3* (1.4 μmol/g), and *TaNRX1-OE-6* (1.3 μmol/g)] was significantly (*P* < 0.05) lower than that in the leaves of the WT (2.5 μmol/g) (Figure 6B). The results of the NBT staining of leaves was similar to those of DAB staining (Figure 6C), and the accumulation of O_2_^–^ in the leaves of the OE lines [*TaNRX1-OE-1* (83.9 nmol/g), *TaNRX1-OE-3* (80.6 nmol/g), and *TaNRX1-OE-6* (85.6 nmol/g)] was significantly (*P* < 0.01) lower than that in the leaves of the WT (103.6 nmol/g); however, the RNAi lines showed the opposite results (Figure 6D).

Superoxide dismutase, CAT, and POD are important antioxidant enzymes in plants and are vital for scavenging ROS. There were no significant differences in SOD, CAT, and POD activities under normal conditions in wheat leaves. After 10 days of drought stress, the activities of SOD, CAT, and POD enzymes were significantly (*P* < 0.05) higher than those in the WT, and activities of SOD, CAT, and POD enzymes in RNAi lines were lower than that of WT, but the difference was not significant (Figures 6E–G). Thus, *TaNRX1* OE was related to the increase of antioxidant enzymes activity and reduced the accumulation of ROS under drought stress.

**FIGURE 7:**
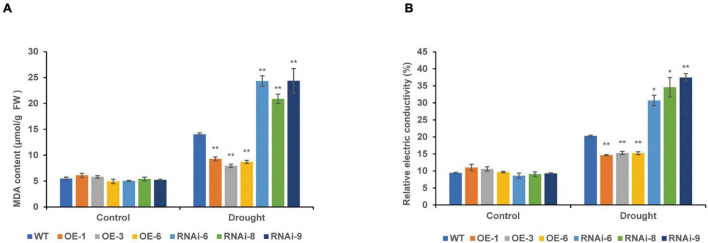
Effect of drought stress on the malondialdehyde and relative electrical conductivity of WT and transgenic wheat. **(A)** Malondialdehyde content of 4 weeks WT and *TaNRX1* transgenic wheat in normal water supply and 10 days of drought stress (*n* = 4). **(B)** Relative electrical conductivity (*n* = 4). Data represent the mean ± SD. WT, wild type; OE-1–OE-6, *TaNRX1* overexpression T_3_ homogeneous lines; RNAi-6–RNAi-9, *TaNRX1* RNA interference T_3_ homogeneous lines. *P < 0.05, **P < 0.01 represent the significant difference between the transgenic line and the WT, respectively.

Under normal conditions, there were no significant differences in the MDA content and REC between the WT and transgenic wheat leaves. After 10 days of drought stress, the MDA content of the RNAi lines [*TaNRX1-RNAi-6* (24.3 μmol/g), *TaNRX1-RNAi-8* (20.8 μmol/g), and *TaNRX1-RNAi-9* (24.4 μmol/g)] was the highest, followed by the WT (14.0 μmol/g). The MDA content of the OE lines [*TaNRX1-OE-1* (9.3 μmol/g), *TaNRX1-OE-3* (7.9 μmol/g), and *TaNRX1-OE-6* (8.7 μmol/g)] was the lowest, with significant differences in the WT and transgenic wheat lines. Changes in REC and MDA content showed similar results (Figures 7A,B).

### RNA-Seq

**FIGURE 8:**
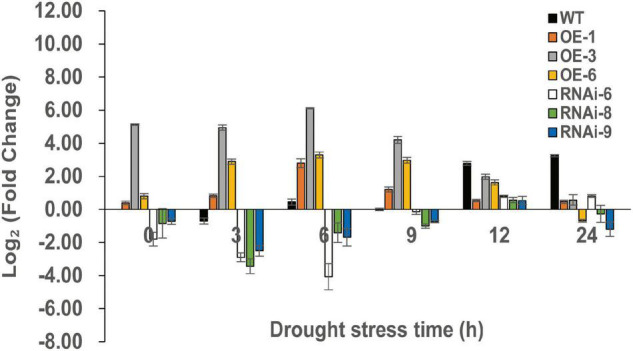
Relative expression of *TaNRX1* in different lines of transgenic wheat under different drought stress times. Data represent the mean ± SD (*n* = 3). WT, wild type; OE-1–OE-6, *TaNRX1* overexpression T_3_ homogeneous lines; RNAi-6–RNAi-9, *TaNRX1* RNA interference T_3_ homogeneous lines.

To identify DEGs after *TaNRX1* OE or RNAi in wheat, we performed RNA-seq. RT-qPCR results showed that the OE (*TaNRX1-OE-3*) and RNAi (*TaNRX1-RNAi-6*) lines had the highest and lowest relative expression levels of *TaNRX1* after 6 h of drought stress, respectively (Figure 8). Therefore, 2-week-old WT, *TaNRX1-OE-3*, and *TaNRX1-RNAi-6* plants under drought stress (0 and 6 h) were selected for RNA-seq.

The RNA-seq analysis of 18 samples of WT and transgenic wheat produced 211.93 Gb of clean data, before and after drought stress; the clean data of each sample reached 10.03 Gb, and the percentage of the Q30 base was 93.74% or above. The sequences of the raw reads have been deposited in the NCBI Sequence Read Archive (SRA) under accession numbers SRR16077951, SRR16077950, SRR16077959, SRR16077958, SRR16077957, SRR16077956, SRR16077955, SRR16077954, SRR16077953, SRR16077952, SRR16077949, SRR16077948, SRR16077965, SRR16077964, SRR16077963, SRR16077962, SRR16077961, and SRR16077960 at the SRA (Sequence Read Achieve) of NCBI. Clean reads of each sample were mapped to a reference genome, and the alignment efficiency ranged from 91.09 to 93.95%. The GC content in all samples exceeded 50%, indicating that the RNA-seq data were highly reliable and could be used for subsequent experimental analysis.

Pearson’s correlation coefficient (*r*) was used as an evaluation index for biological repeat correlation. The closer *r*^2^ is to 1, the stronger the correlation between the two duplicated samples. Sample correlation results in this experiment are shown in Supplementary Figure 3. One of the three biological replicates of each sample showed a low correlation. After removing replicates with a low correlation, DEGs were identified according to the differing expression levels of genes in different samples, and functional annotation and enrichment analyses were conducted.

The number of DEGs identified in different RNA-seq comparisons is shown in Supplementary Figure 4. In total, 11,692 genes were differentially expressed, of which 7,858 were upregulated and 3,834 were downregulated in the WT after drought stress compared with those under pre-drought stress. In total, 9,140 genes were differentially expressed, with 5,765 being upregulated and 3,375 downregulated in the OE lines. The RNAi lines had a total of 9,725 DEGs, with 4,342 being upregulated and 5,383 downregulated. Compared with the WT before drought stress, the OE lines had a total of 7,458 DEGs, with 3,770 being upregulated and 3,688 downregulated. Compared with the WT, the RNAi lines had a total of 8,942 genes that were differentially expressed, with 5,789 being upregulated and 3,153 downregulated under control conditions. Compared with the WT after drought stress, the OE lines had 427 DEGs, of which 203 were upregulated and 224 downregulated. Compared with the WT, the RNAi lines had 3,275 genes that were differentially expressed, with 586 being upregulated and 2,689 downregulated after drought stress.

In order to identify the DEGs specifically affected by *TaNRX1*, the DEGs in WT, *TaNRX1* OE, and RNAi wheat were compared before and after drought stress. We removed the DEGs shared with the WT, and a total of 1,991 common DEGs were identified in the transgenic wheat (Supplementary Figure 5). DEGs were verified using RT-qPCR analysis. To this end, we randomly selected seven DEGs, including triphosphate isomerase, heat shock protein 70, and chlorophyll a/b binding protein. The gene expression trend was consistent with the RNA-seq results (Supplementary Figure 6), which strongly indicated the reliability of the RNA-seq data for use in subsequent analysis.

**FIGURE 9:**
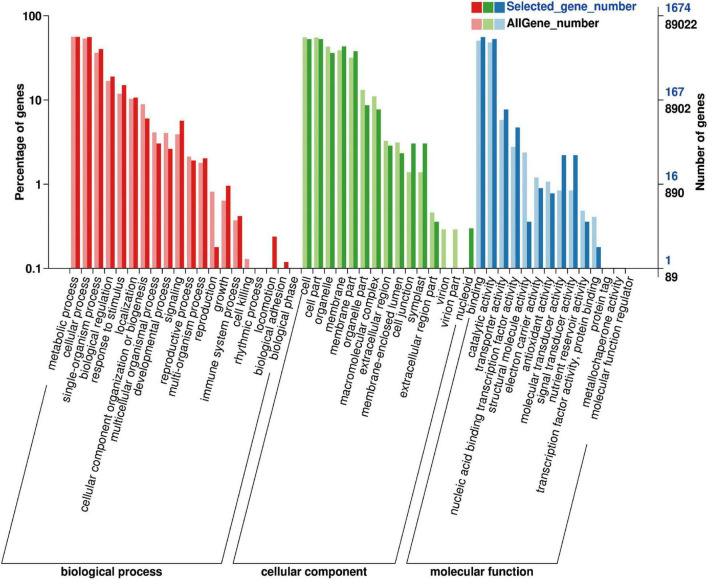
Biological function GO enrichment analysis of 1,991 differentially expressed genes.

The 1,991 DEGs were further analyzed using GO and KEGG. GO analysis described the biological components, cellular components, and molecular functions of the genes. Among the biological processes, DEGs were mainly concentrated in metabolic, cellular, single-organism processes; biological regulation; response to stimulus; and localization. Among the cell components, DEGs were mainly distributed in cells, membranes, and organelles. The molecular functions of the DEGs included binding ability, catalytic activity, transporter activity, nucleotide binding transcription factor activity, molecular transducer activity, and signal transducer activity (Figure 9).

**FIGURE 10:**
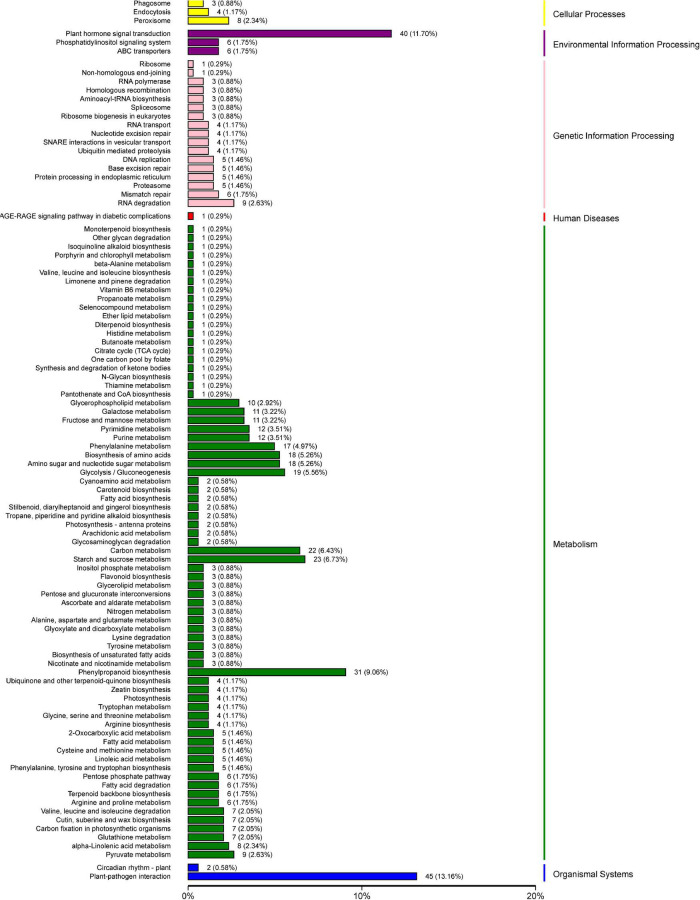
Physiological and biochemical metabolic pathway KEGG enrichment analysis of 1,991 differentially expressed genes.

To further explore the metabolic pathways involved in the 1,991 DEGs, the DEGs were assigned to 71 KEGG pathways. The DEGs were mainly distributed in the plant–pathogen interaction (45 genes), plant hormone signal transduction (40 genes), phenylpropanoid biosynthesis (31 genes), starch and sucrose metabolism (23 genes), carbon metabolism (22 genes), glycolysis and gluconeogenesis (19 genes), amino sugar and nucleotide sugar metabolism (18 genes), and biosynthesis of amino acids (18 genes) pathways (Figure 10).

## Discussion

### *TaNRX1* Positively Regulates Drought Tolerance of Wheat

Nucleoredoxin belongs to the TRX superfamily, which is widely distributed in organisms. It performs the function of electron transfer, which is involved in a variety of life activities (Montrichard et al., 2009; Urbainsky et al., 2018). To date, many studies have been conducted on the biological function of the NRX gene, but information on the specific mechanism of how the gene participates in the response to abiotic stress in wheat is still very limited.

In this study, we selected three wheat lines with *TaNRX1* OE (*TaNRX1-OE-1*, *TaNRX1-OE-3*, and *TaNRX1-OE-6*) and three wheat lines with *TaNRX1* knockdown (*TaNRX1*-*RNAi-6*, *TaNRX1*-*RNAi-8*, and *TaNRX1*-*RNAi-9*) to study the function of this gene in the drought tolerance of wheat. The results showed that the *TaNRX1-OE* lines had relatively better growth and development than those of the WT and *TaNRX1-RNAi* lines at both the seedling and heading stages under drought stress conditions (Figures 3A, 4A), and that the survival rate of *TaNRX1-OE* lines was higher after rehydration after drought stress at the seedling stage (Figure 3B). Compared with WT, *TaNRX1-OE* lines had more dry matter content of roots, longer root length and stronger root activity (Figures 2B–E), suggesting that *TaNRX1-OE* lines might have stronger water absorption capacity. Drought stress can change the chlorophyll content in plant leaves. It is generally believed that when the degree of drought stress approaches the critical state of plants, chlorophyll begins to degrade and thus its content decreases (Tohru et al., 2001). In this study, following drought stress experiments, the chlorophyll content of *TaNRX1-OE* lines decreased by the same level or at a slightly lower level than that in the WT (Figure 4B), suggesting that *TaNRX1-OE* lines can tolerate a higher degree of drought stress than the WT. In addition, as photosynthesis is key to the growth and development of crops as well as yield and quality, a change in water status becomes an important limiting factor affecting the photosynthetic activities of crops (Guo et al., 2013). In this study, *TaNRX1-OE* lines exhibited better photosynthetic performance (Figures 4C–F). Under drought treatment, the Pn content of leaves decreased slightly, suggesting that *TaNRX1* OE improved the physiological activity intensity in wheat, enhanced the ability to capture external CO_2_, and led to an increase in photosynthetic intensity. In *TaNRX1*-*OE* lines, the Gs in leaves was relatively high, which could decrease CO_2_ diffusion resistance, facilitate CO_2_ exchange between the stomata and the outside world, and promote an increase in Pn content. The Tr of leaves in *TaNRX1-OE* lines was small, suggesting that *TaNRX1* OE reduced transpiration and water loss in leaves to a certain extent to maintain the normal physiological activities of plants. These results suggest that under drought stress, the carbon assimilation capacity of *TaNRX1*-*OE* lines was significantly improved, whereas that of *TaNRX1*-RNAi lines was contrary, suggesting that *TaNRX1* positively regulates the drought tolerance of transgenic wheat.

### Plant–Pathogen Interaction Pathway Mediates the Resistance of *TaNRX1* Transgenic Wheat to Drought Stress

The DEGs in the plant–pathogen interaction pathway mainly included Ca^2+^ signal transduction-related genes and *NBS-LRR* genes. As sessile organisms, plants often suffer from biotic (necrotrophic and biotrophic pathogens) and abiotic (drought, salt, heat, or cold) stresses in the natural environment. Several studies have shown that the defense mechanisms of these two forms of stress in plants are related; that is, some stress resistance genes can respond to both biotic and abiotic stresses simultaneously (Atkinson and Urwin, 2012; Ku et al., 2018). Among the many signal transduction pathways in plants, Ca^2+^ is a multifunctional second messenger. The change in Ca^2+^ concentration inside and outside the cell is an important part of the signal transduction pathway of plants in response to growth, development, and environmental stimuli. Three types of Ca^2+^ signaling systems have been identified, which include calmodulin, calmodulin-like protein (CML), calcium-dependent protein kinase, and calcineurin B-like protein. CMLs are involved in various plant growth and development processes, hormone-regulated cell activities, and related defense mechanisms induced by pathogens and inducers. They are induced by various stresses (Bender and Snedden, 2013). CMLs are sensitive to abiotic stresses such as abscisic acid (ABA), methyl jasmonate, and drought and salt stresses (Mccormack et al., 2005). Magnan et al. (2008) found that water shortage treatment induced a rapid and transient increase in the expression level of *AtCML9.* The expression level increased rapidly by seven times within 10 min, which suggests that *AtCML9* is associated with the improvement of water deficit tolerance. *CML20* is a negative regulator of ABA-induced stomatal movement in *Arabidopsis*. The loss of *CML20* resulted in a smaller stomatal aperture and less water loss in the leaves of the *CML20* gene deletion mutant than in the WT (Wu et al., 2017). Compared with WT tomato plants, tomato plants overexpressing *ShCML44* have lower accumulation of MDA and a lower degree of membrane damage under cold and drought stress. The activities of antioxidant enzymes, gas exchange, and water retention were enhanced. The loss of *CML20* also showed a decrease in ROS levels and an increase in the RWC (Munir et al., 2016).

NBS-LRR proteins containing nucleotide binding sites (NBSs) and leucine-rich repeats (LRRs) are the main immune receptors in plants. In total, 413 NBS resistance genes of orchardgrass (*Dactylis glomerata* L.) were studied; among these, 11 were differentially expressed under waterlogging stress, 5 were differentially expressed under waterlogging and drought stress, and 1 was differentially expressed under waterlogging and heat stress, suggesting that some *NBS-LRR* genes also respond to abiotic stress while defending against pathogen infection (Ren et al., 2020).

In the RNA-seq results of this study, the 1,991 DEGs before and after drought stress treatment mainly included *TaCML10*, *TaCML25*, *TaCML26*, *TaCML27*, *TaCML30*, *TaCML31*, and the NBS-LRR protein gene *TaRPM1* in the plant–pathogen interaction pathway. ROS accumulate under abiotic stress conditions and are considered an important index of plant tolerance to abiotic stress (You and Chan, 2015; Xu et al., 2018). According to the results of previous studies, it was speculated that after the overexpression of *TaNRX1*, the changes in the expression levels of these DEGs improve the drought resistance of transgenic wheat by mediating the changes in ABA content and the removal of ROS. The DAB and NBT staining results of the leaves of *TaNRX1-OE* and *TaNRX1-RNAi* lines showed that the ROS in wheat lines overexpressing *TaNRX1* were effectively removed (Figures 6A,C). The higher leaf RWC and the lower water loss rate of isolated leaves in *TaNRX1-OE* lines suggested that the stomatal closure of wheat leaves increased after *TaNRX1* OE, whereas *TaNRX1-RNAi* lines showed the opposite result (Figures 5A,B). The results of these physiological indices were consistent with our prediction above.

### The Plant Hormone Signal Transduction Pathway Affects the Resistance of Transgenic Wheat With *TaNRX1* Gene to Drought Stress

The DEGs in plant hormone signal transduction pathways mainly include genes related to ABA hormone and cytokinin (CTK) hormone signal transduction. Protein phosphatase 2C (PP2C) is a monomer serine/threonine protein phosphatase dependent on Mg^2+^ or Mn^2+^ (Cohen, 1989; Stern et al., 2007). PP2C is widely involved in various ABA signaling pathways in higher plants, including ABA-induced seed germination and dormancy, regulation of guard cells and ion channels, stomatal closure, and biological and abiotic stress. Liu et al. (2012) reported an *Arabidopsis* gene *AtPP2CG1*, which encodes a protein belonging to the G subgroup of the PP2C family and regulates the response of *Arabidopsis* to salt stress, with its regulation being ABA dependent. The RNA-seq results in this study showed that the expression levels of *TaPP2C37*, *TaPP2C50*, and *TaPP2C68* were all increased in *TaNRX1-OE*, *TaNRX1-RNAi*, and WT lines after drought treatment compared with those at pre-drought treatment, and the increased expression was higher in *TaNRX1-OE* lines.

Cytokinins also play an important role in plant resistance to abiotic stress. The CTK signal is mediated by the CTK receptor histidine kinase (HK), histidine-containing phosphotransfer (HPT), and a complex two-component system (TCS) consisting of an effector response regulator (RR) (Hwang and Sheen, 2001; Hutchison and Kieber, 2002). Jain et al. (2006) systematically studied the expression of A-type *RR* genes in different organs under different exogenous hormone treatments and under different environmental stresses by quantitative PCR. The results showed that most *RR* genes were induced by CTK expression. In addition, the expression level of *OsRR6* was significantly increased under high salinity, water shortage, and low temperature stresses. The RNA-seq results in this study showed that the expression levels of *TaHPT4*, *TaHPT5*, and *TaR22* were all increased in the three lines after drought treatment compared with those at pre-drought treatment, and the extent of upregulation was higher in *TaNRX1-OE* lines. These results suggest that *TaNRX1* could improve the drought resistance of *TaNRX1-OE* lines by mediating the expression of TCS-related genes and regulating the CTK signaling pathway. In this study, the chlorophyll content of *TaNRX1-OE* lines decreased by the same level or at a slightly lower level than that in the WT after drought stress (Figure 4B). However, CTKs could inhibit the decomposition of chlorophyll (Bajguz and Piotrowska-Niczyporuk, 2014; Dobránszki and Mendler-Drienyovszki, 2014), which confirmed that *TaNRX1* mediates the CTK signaling pathway.

### Phenylalanine Metabolism Pathway Is Associated With the Resistance of Transgenic Wheat With *TaNRX1* Gene to Drought Stress

Under different abiotic stresses, plants maintain their performance using various methods. One is to improve secondary metabolism, as the accumulation of secondary metabolites helps plants to alleviate the damage caused by various stresses (Sharma et al., 2012; Shahzad et al., 2018). Phenylpropane-like metabolism is a typical representative of plant secondary metabolism. Under drought and other adverse conditions, the metabolic processes of phenylpropanes actively participate in the stress resistance of plants. The metabolites in the reaction process not only stabilize cell osmotic potential and cell structure or avoid cell collapse and metabolic disorders caused by cell dehydration, they also eliminate oxidative damage to plants by promptly removing the ROS produced during the stress resistance process.

Isoprene production in transgenic tobacco maintains plant photosynthesis by affecting phenylpropane metabolism and protects plants from the effects of drought stress (Tattini et al., 2014). Transcriptome analysis revealed that the metabolism of hormones and ROS as well as cell wall biosynthesis, especially phenylpropane-like metabolism, promotes root growth in wheat under drought conditions (Dalal et al., 2018). Herrero et al. (2013) showed that *AtPOD72* is involved in lignin formation, and Fernandez-Pérez et al. (2015) showed that inhibition of the last step of lignin biosynthesis affects whole phenylpropanol biosynthesis. The RNA-seq results of this study showed that the expression levels of *Ta*β*-Glu12*, *TaPOD5*, *TaPOD11*, and *TaPOD55* in *TaNRX1-OE*, *TaNRX1-RNAi*, and WT lines were all increased after drought treatment, and the extent of upregulation was greater in *TaNRX1-OE* lines. These results suggest that *TaNRX1* may enhance drought resistance in wheat by mediating phenylpropane metabolism.

### Multiple Transcription Factors Are Related to the Resistance of Transgenic Wheat With *TaNRX1* Gene to Drought Stress

Transcription factors are a class of important regulatory proteins that play an important and unique role in regulating plant growth and development and are involved in most life processes in cells. Transcription factors regulate gene transcription or termination through specific binding with nucleotide sequences in the promoter region of target genes, and act as important “switches” for downstream target genes, thus generating a defense response. In addition, they realize their biological functions by interacting with a variety of proteins (Brunelle and Chua, 1993). In this study, 1,991 DEGs contained a large number of transcription factor genes, among which the frequency of *WRKY*, *MYB*, and *bHLH* genes was the highest.

WRKY proteins are involved in plant abiotic stress responses through complex signal transduction pathways and play an important regulatory role. Drought-tolerant plants can improve their drought resistance by accumulating large amounts of sucrose, tetrose, and a series of raffinose family oligosaccharides. In particular, WRKY proteins are involved in drought responses in plants through glucose metabolism pathways (Wu et al., 2009) and can directly regulate the expression of drought resistance genes to improve drought resistance in plants. For example, WRKY transcription factors can regulate the expression of ABA-related genes and participate in ABA-mediated drought response pathways. Under drought stress conditions, ABA levels in plants usually increase and the stomata of the leaves are closed to maintain water in the cells (Sakamoto, 2004). In addition to directly regulating the expression patterns of target genes, some WRKY proteins indirectly regulate the expression levels of functional genes by interacting with other transcription factors (Wei et al., 2008). AtWRKY53 OE in *Arabidopsis* inhibits stomatal closure by reducing the hydrogen peroxide content in guard cells and accelerating starch metabolism, thereby negatively regulating drought (Sun and Yu, 2015). Cotton *GhWRKY41* positively regulates plant salt tolerance and drought resistance by regulating stomatal closure and expression of antioxidant-related genes (Chu et al., 2015). *AtWRKY46*, *AtWRKY54*, and *AtWRKY70* positively regulated growth and development and negatively regulated the drought stress response in *A. thaliana* (Chen et al., 2017). In this study, the RNA-seq results showed that the expression levels of *TaWRKY53* and *TaWRKY70-like* were decreased in the *TaNRX1-OE* lines after drought stress treatment compared with that at pre-drought stress, whereas the increase in expression levels of *TaWRKY41* was higher in the *TaNRX1-OE* lines. These findings are similar to those of the previous studies on *A. thaliana* and cotton, as described above.

The MYB transcription factor family is named by its conserved MYB domain and is present in all eukaryotes. MYB transcription factors not only respond to drought and other abiotic stresses by inducing stress-related gene expression and stomatal opening and closing, but also regulate phenylpropane-like metabolic pathways to regulate plant growth, development, and stress tolerance. The wheat MYB transcription factor TaODORANT1 positively regulates the response of transgenic tobacco to drought and salt stress (Wei et al., 2017). In this study, RNA-seq results showed that the expression of wheat MYB transcription factor gene *TaODORANT1* was higher in *TaNRX1-OE* lines after drought stress than under pre-drought stress.

Basic/helix-loop-helix (bHLH) is a type of transcription factor that contains a basic/helix-loop-helix structure, is widely present in eukaryotes, and comprises many members. The expression of bHLH transcription factor OsbHLH148 in rice is significantly increased under drought conditions. OsbHLH148 participates in regulating the expression of genes related to the jasmonic acid signaling pathway, thereby increasing the drought tolerance of plants. In addition, another bHLH transcription factor RERT1 (OsbHLH006) in rice is also involved in mediating the jasmonic acid signal pathway and drought stress response (Seo et al., 2011). *NtbHLH123* overexpression in plants shows lower electrolyte leakage under cold stress, reducing the content of malondialdehyde and the accumulation of reactive oxygen species (ROS), thereby alleviating the cold stress-induced oxidative damage of cell membranes (Zhao et al., 2018). The heterologous expression of the gene AabHLH35 of *Anthurium andraeanum* in *Arabidopsis* can improve the cold and drought tolerance of transgenic *Arabidopsis* species (Jiang et al., 2019). In this study, RNA-seq results showed that the expression of wheat bHLH transcription factor gene *TabHLH35* was higher in *TaNRX1-OE* lines after drought stress than under pre-drought stress.

bZIP-type transcription factors recognize *cis*-acting elements whose core sequence is ACGT, including CACGTG (G box), GACGTC (C box), and TACGTA (A box), and the promoter regions of some genes induced by light or ABA containing these elements (Nakagawa et al., 1996). ABA and abiotic stress induce ABF/AREB expression, and ABA induces AREB1/2 phosphorylation. This phosphorylation is necessary for downstream gene induction by ABRE1/2 and could occur at the phosphorylation site of casein kinase II in the conserved domain. Kinase activates the bZIP protein through phosphorylation so that it can bind to the ABRE element in the promoter region of the gene, resulting in the expression of downstream genes regulated by ABA (Schlögl et al., 2008). Zong et al. (2016) found that *OsbZIP23* can positively regulate the expression of *OsNCED4* by directly binding to the promoter of *OsNCED4.* It can then positively regulate the content of ABA in rice, thus further activating ABA signal transduction. The RNA-seq results in this study showed that the expression levels of wheat bZIP transcription factor genes *TabZIP23* and *TaTRAB1* were higher in *TaNRX1-OE* lines after drought stress than under pre-drought stress. It was speculated that *TaNRX1* OE in wheat mediated ABA signal transduction by affecting the expression of wheat bZIP transcription factor genes, thereby enhancing the drought resistance of wheat.

### Some Proteins Encoded by Differentially Expressed Genes Are Potential Target Proteins of *TaNRX1*

Thioredoxin family members are molecular redox switches in organisms. They reduce the target protein through the transfer of electrons, thereby changing the catalytic activity or binding ability of the target protein, which in turn affects more biological functions (Collin et al., 2003). Montrichard et al. (2009) reviewed approximately 500 established and potential target proteins of TRX in terrestrial plants and aerobic microorganisms. Urbainsky et al. (2018) used a differential thiol-labeling technique and quantitative mass spectrometry to identify 609 NRX target proteins in the neuronal cells of mice. The biological processes and molecular functions of these interacting proteins were analyzed, and the results show that NRXs have multiple functions in the redox regulation of metabolic pathways, cell morphology, and signal transduction. Compared with previous studies, we found that some of the proteins encoded by the 1,991 common DEGs identified in this study, such as enzymes involved in the glycolysis/gluconeogenesis pathway and aldehyde dehydrogenases (ALDHs) were also target proteins of TRX or NRX. Glycolysis uses many enzymes to convert glucose into pyruvate and produce ATP, and the activation of genes related to glycolysis and gluconeogenesis may play a role in the drought response of plants. When plants are subjected to abiotic stresses, such as drought, the ATP-generating reaction catalyzed by pyruvate kinase is inhibited. As a glycolytic enzyme, pyruvate, phosphate dikinase (PPDK) can bypass the reaction catalyzed by pyruvate kinase and produce two ATP equivalents, which is very important for plants to cope with stress (Plaxton and Tran, 2011). Sucrose synthase (SUS) is a key enzyme involved in sucrose metabolism. As SUS requires less energy to catalyze sucrose decomposition, plants tend to rely more on SUS to provide reducing sugar when under stress and with limited energy supply to maintain normal growth and development (Fu and Park, 1995; Ricard et al., 1998; Biemelt et al., 1999). Soluble sugars play an important role in stress resistance in plants. They facilitate the plant to absorb water better by maintaining the osmotic pressure balance in the cell so that the cell can resist the adverse environment. The results of this study show that after drought stress, *TaNRX1-OE* lines had higher contents of proline and soluble sugar in the leaves compared with those in the WT and *TaNRX1-RNAi* lines, which resulted in higher leaf RWC and a lower leaf water loss rate *in vitro*. Water retention and water holding capacity of transgenic wheat leaves with the *TaNRX1* gene were enhanced under drought stress. The RNA-seq results showed that genes involved in the glycolysis/gluconeogenesis pathway, including *TaACS, TaPGM, TaPFP, TaSUS*, and *TaTPI*, which are the encoding genes of acetyl-CoA synthase, phosphoglucose transgenic enzyme, pyrophosphate: fructose 6-phosphate 1-phosphate transferase, sucrose synthase and propanones phosphate isomerase, respectively, were all more upregulated in *TaNRX1-OE* lines than in the WT and *TaNRX1-RNAi* lines, which is consistent with the results of the above physiological indicators.

Reactive oxygen species can promote the peroxidation of membrane lipids to cause chemical reactions that generate corresponding aldehydes and cause the accumulation of aldehydes in the organism. Excessive aldehydes react with proteins and nucleic acids, destroying the normal structure and function of proteins and nucleic acids. The total amount of MDA determines the level of lipid peroxidation in plant cells (Perozich et al., 2010). ALDHs are a large class of NAD(P)^+^-dependent catalytic enzymes that can combine NAD^+^ or NADP^+^ to catalyze the oxidative dehydrogenation of endogenous or exogenous aldehydes to generate corresponding carboxylic acids (Rodrigues et al., 2006), thereby reducing the accumulation of aldehydes in plants and achieving detoxification. Thus, ALDHs can alleviate the toxicity of plants under abiotic stress conditions and improve the ability of plants to tolerate adversity. In this study, the MDA content and REC of leaves of *TaNRX1-OE* lines were significantly lower than those of the WT and *RNAi* lines after drought stress, which is consistent with the changes in aldehyde dehydrogenase gene expression before and after drought stress, as indicated by the RNA-seq results.

## Conclusion

We demonstrated that *TaNRX1* positively regulates drought resistance in wheat. *TaNRX1* affected the expression of genes related to drought stress through transcriptomics, with these DEGs being mainly distributed in plant–pathogen interactions, plant hormone signal transduction, phenylpropane biosynthesis, starch and sucrose metabolism, and carbon metabolism pathways. This study could provide a new sight for the analysis of drought resistance mechanism in wheat and a valuable target for the production of drought-resistant wheat varieties.

## Data Availability Statement

The sequences of the raw reads have been deposited in the NCBI Sequence Read Archive (SRA) under accession numbers SRR16077951, SRR16077950, SRR16077959, SRR16077958, SRR16077957, SRR16077956, SRR16077955, SRR16077954, SRR16077953, SRR16077952, SRR16077949, SRR16077948, SRR16077965, SRR16077964, SRR16077963, SRR16077962, SRR16077961, and SRR16077960.

## Author Contributions

XZ designed the experiments with the help of GX. YZ, and JZ performed the experiments and wrote the manuscript. FW, TS, YY, MY, QF, and YNY provided assistance with the experiments. All authors contributed to the article and approved the submitted version.

## Conflict of Interest

The authors declare that the research was conducted in the absence of any commercial or financial relationships that could be construed as a potential conflict of interest.

## Publisher’s Note

All claims expressed in this article are solely those of the authors and do not necessarily represent those of their affiliated organizations, or those of the publisher, the editors and the reviewers. Any product that may be evaluated in this article, or claim that may be made by its manufacturer, is not guaranteed or endorsed by the publisher.

## Footnotes

1
www.biocloud.net

## Supplementary Material

Supplementary Figure 1 The PCR detection for T_1_–T_3_ generations of transgenic wheat. WT, wild type; 1–11, transgenic wheat T_1_–T_3_ independent lines; M, 2,000 bp marker.

Supplementary Figure 2 The relative expression of *TaNRX1* in transgenic wheat after drought 10 and 14 days (*n* = 3). Data represent the mean ± SD. WT, wild type; OE-1–OE-6, *TaNRX1* overexpression T_3_ homogeneous lines; RNAi-6–RNAi-9, *TaNRX1* RNA interference T_3_ homogeneous lines. **P* < 0.05, ***P* < 0.01 represent significant difference between the transgenic line and the WT, respectively. The 2^–ΔΔ^ *^*C*^*^*T*^ method was used to calculate the relative expression levels of genes.

Supplementary Figure 3 Heat map of correlation between samples. W01–W03, wild type drought stress 0 h three biological duplicates; W61–W63, wild type drought stress 6 h three biological duplicates; E01–E03, *TaNRX1-OE-3* drought stress 0 h three biological duplicates; E61–E63, *TaNRX1-OE-3* drought stress 6 h three biological duplicates; R01–R03, *TaNRX1-RNAi-6* drought stress 0 h three biological duplicates; R61–R63, *TaNRX1-RNAi-6* drought stress 6 h three biological duplicates.

Supplementary Figure 4 The bar graph of WT, *TaNRX1* overexpression and RNA interference wheat lines differentially expressed genes after PEG6000 treatment 0 and 6 h. DEG set, the name of the differentially expressed genes set; DEG number, the number of differentially expressed genes; up-regulated, the number of up-regulated genes; down-regulated, the number of down-regulated genes. W0, wild type drought stress 0 h; W6, wild type drought stress 6 h; E0, *TaNRX1-OE-3* drought stress 0 h; E6, *TaNRX1-OE-3* drought stress 6 h; R0, *TaNRX1-RNAi-6* drought stress 0 h; R6, *TaNRX1-RNAi-6* drought stress 6 h.

Supplementary Figure 5 Venn diagrams of differentially expressed genes in WT and transgenic wheat lines before and after drought stress. W0, wild type drought stress 0 h; W6, wild type drought stress 6 h; E0, *TaNRX1-OE-3* drought stress 0 h; E6, *TaNRX1-OE-3* drought stress 6 h; R0, *TaNRX1-RNAi-6* drought stress 0 h; R6, *TaNRX1-RNAi-6* drought stress 6 h.

Supplementary Figure 6 RT-qPCR verification of RNA-seq results. **(A)** Relative expression of 70 kDa heat shock protein. **(B)** Relative expression of *WPI6* gene. **(C)** Relative expression of receptor-like protein kinase. **(D)** Relative expression of triosephosphate isomerase. **(E)** Relative expression of chloroplast a/b binding protein. **(F)** Relative expression of protein phosphatase 2C. **(G)** Relative expression of UDP-glucose-6-dehydrogenase. Data represent the mean ± SD (*n* = 3). E0, *TaNRX1-OE-3* drought stress 0 h; E6, *TaNRX1-OE-3* drought stress 6 h; R0, *TaNRX1-RNAi-6* drought stress 0 h; R6, *TaNRX1-RNAi-6* drought stress 6 h. The 2^–ΔΔ^ *^*C*^*^*T*^ method was used to calculate the relative expression levels of genes.

Supplementary Table 1 All primers used in the experiment.

Supplementary Table 2 Nucleoredoxin (NRX) and TRX members off-target RNA-seq data. W01–W02, wild type drought stress 0 h two biological duplicates; R01–R02, *TaNRX1-RNAi-6* drought stress 0 h two biological duplicates.
