# Supplementary figures and images for: Nucleoredoxin Gene TaNRX1 Positively Regulates Drought Tolerance in Transgenic Wheat (Triticum aestivum L.)
Source: Front Plant Sci. 2021 Nov 11;12:756338. doi: 10.3389/fpls.2021.756338 (PMC8632643; doi:10.3389/fpls.2021.756338)

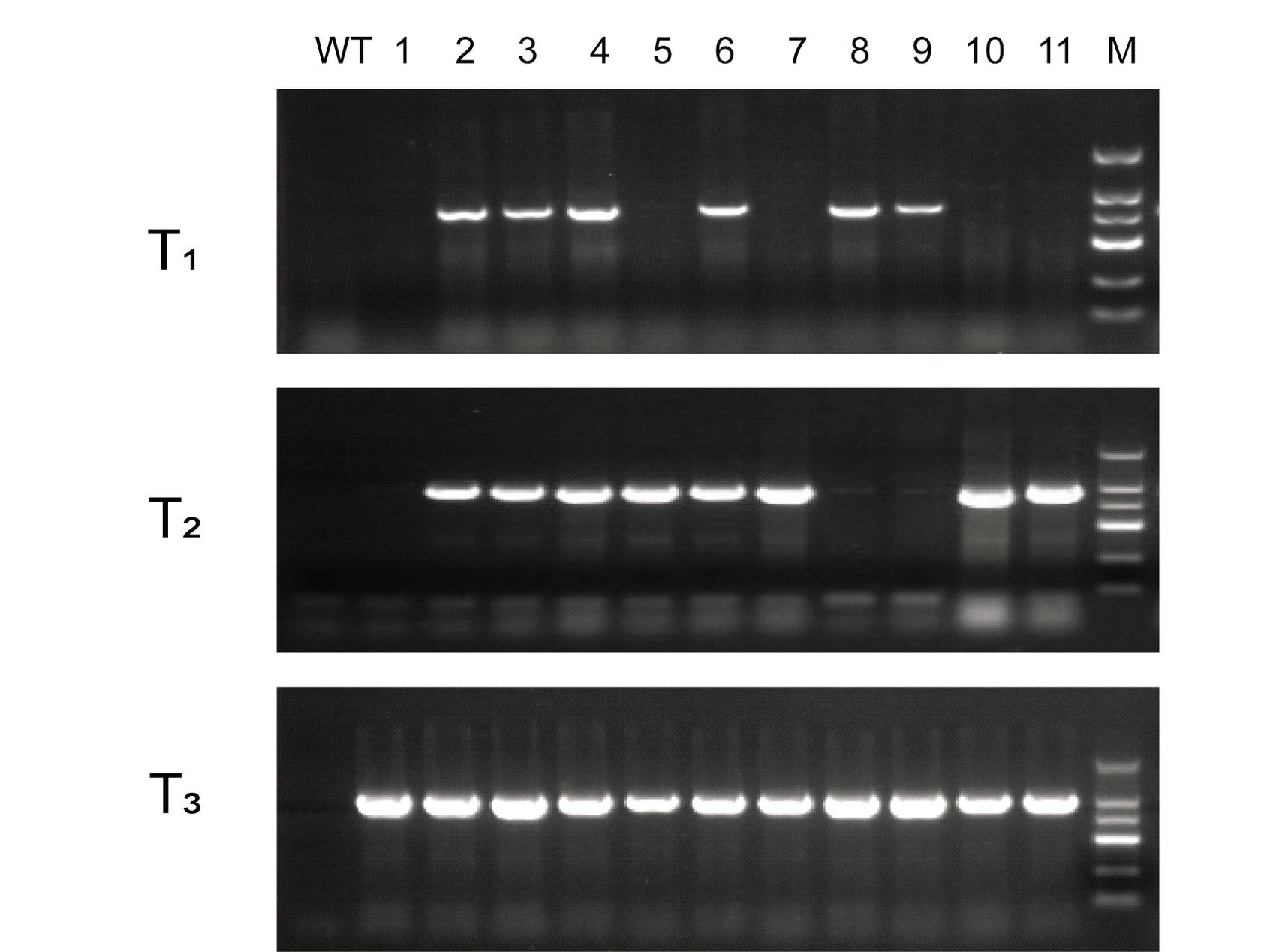

Supplement: Supplementary Figure 1 — The PCR detection for T1–T3 generations of transgenic wheat. WT, wild type; 1–11, transgenic wheat T1–T3 independent lines; M, 2,000 bp marker. [file Image_1.TIF]

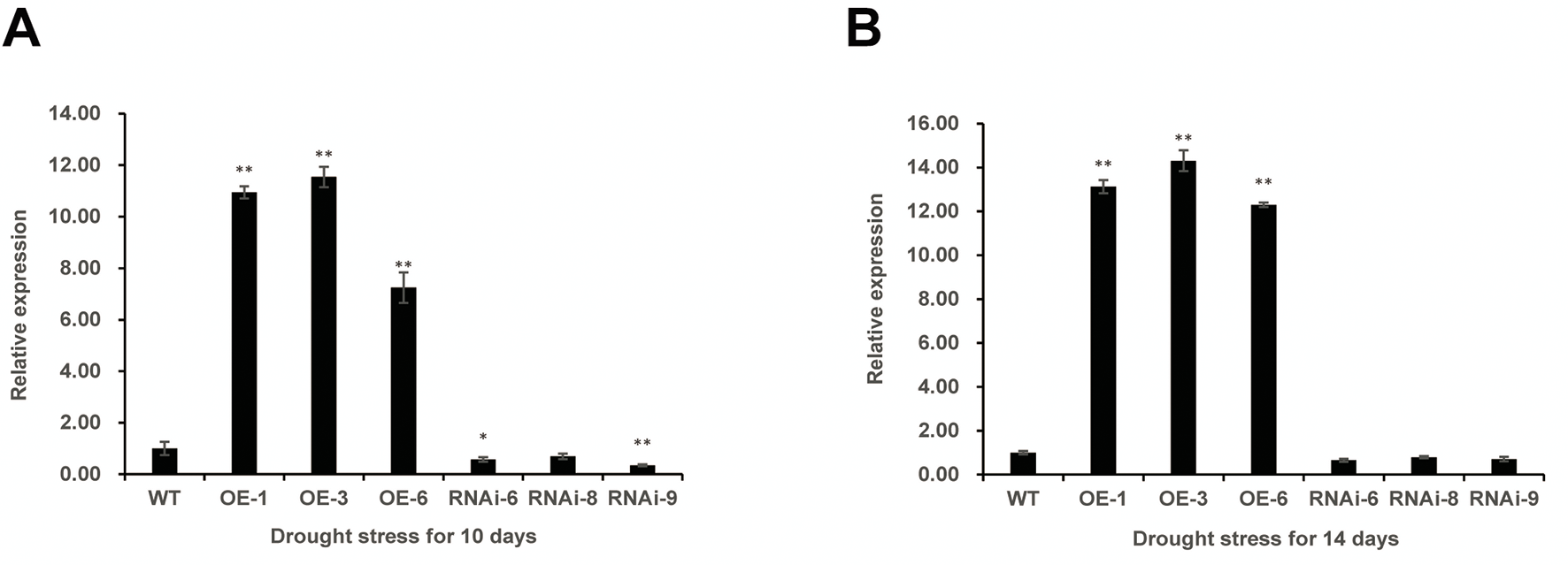

Supplement: Supplementary Figure 2 — The relative expression of TaNRX1 in transgenic wheat after drought 10 and 14 days (n = 3). Data represent the mean ± SD. WT, wild type; OE-1–OE-6, TaNRX1 overexpression T3 homogeneous lines; RNAi-6–RNAi-9, TaNRX1 RNA interference T3 homogeneous lines. *P < 0.05, **P < 0.01 represent significant difference between the transgenic line and the WT, respectively. The 2–ΔΔ CT method was used to calculate the relative expression levels of genes. [file Image_2.TIF]

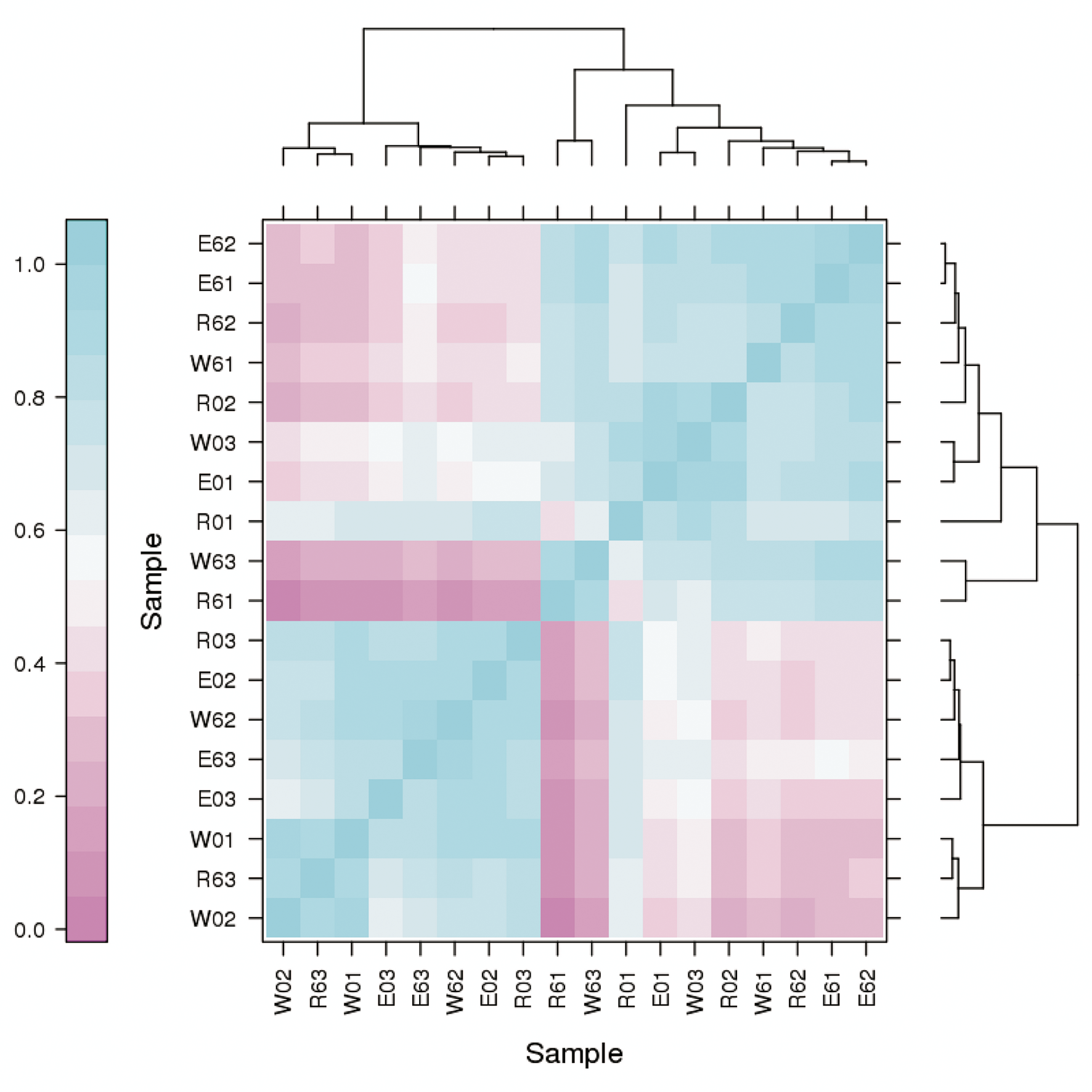

Supplement: Supplementary Figure 3 — Heat map of correlation between samples. W01–W03, wild type drought stress 0 h three biological duplicates; W61–W63, wild type drought stress 6 h three biological duplicates; E01–E03, TaNRX1-OE-3 drought stress 0 h three biological duplicates; E61–E63, TaNRX1-OE-3 drought stress 6 h three biological duplicates; R01–R03, TaNRX1-RNAi-6 drought stress 0 h three biological duplicates; R61–R63, TaNRX1-RNAi-6 drought stress 6 h three biological duplicates. [file Image_3.TIF]

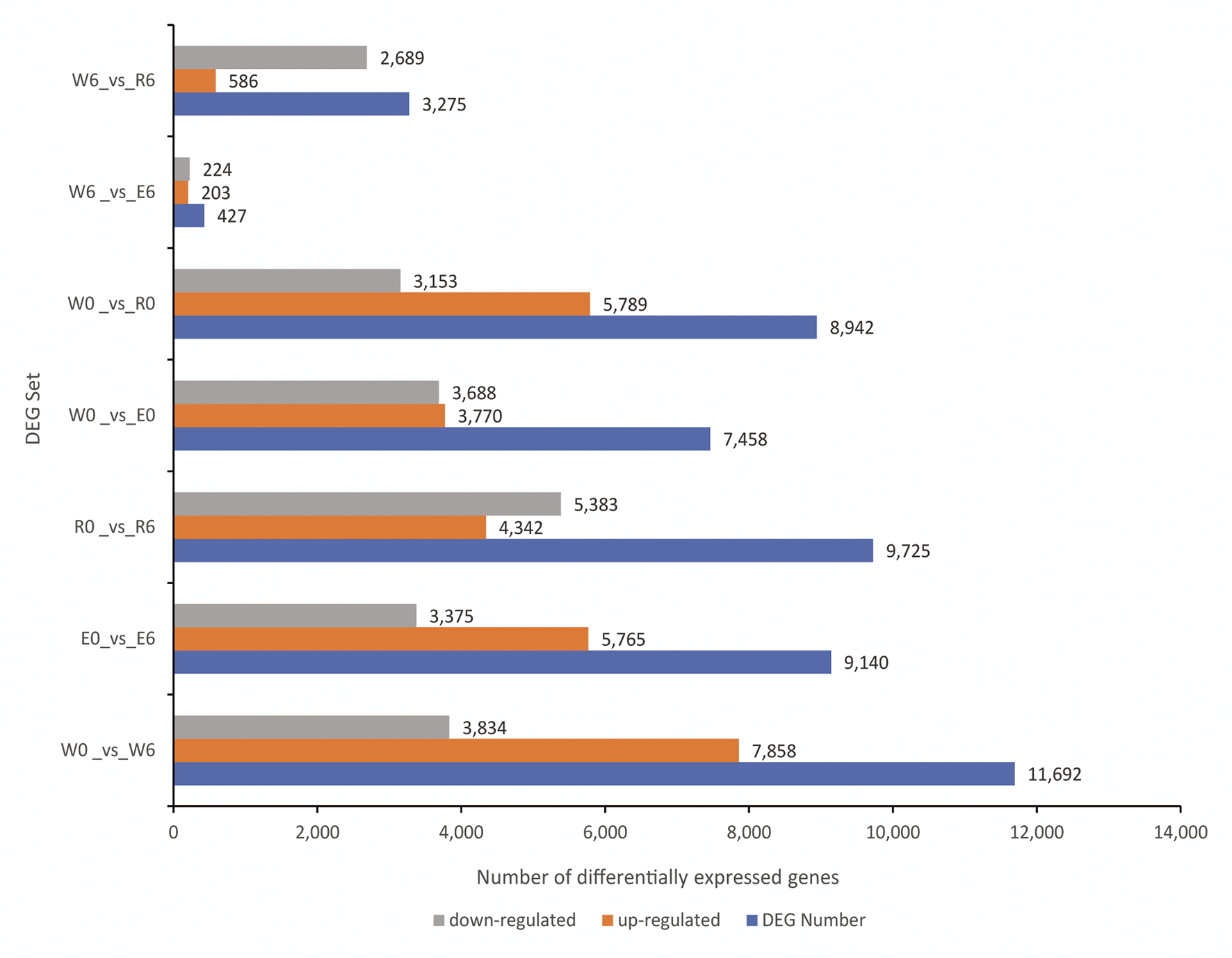

Supplement: Supplementary Figure 4 — The bar graph of WT, TaNRX1 overexpression and RNA interference wheat lines differentially expressed genes after PEG6000 treatment 0 and 6 h. DEG set, the name of the differentially expressed genes set; DEG number, the number of differentially expressed genes; up-regulated, the number of up-regulated genes; down-regulated, the number of down-regulated genes. W0, wild type drought stress 0 h; W6, wild type drought stress 6 h; E0, TaNRX1-OE-3 drought stress 0 h; E6, TaNRX1-OE-3 drought stress 6 h; R0, TaNRX1-RNAi-6 drought stress 0 h; R6, TaNRX1-RNAi-6 drought stress 6 h. [file Image_4.TIF]

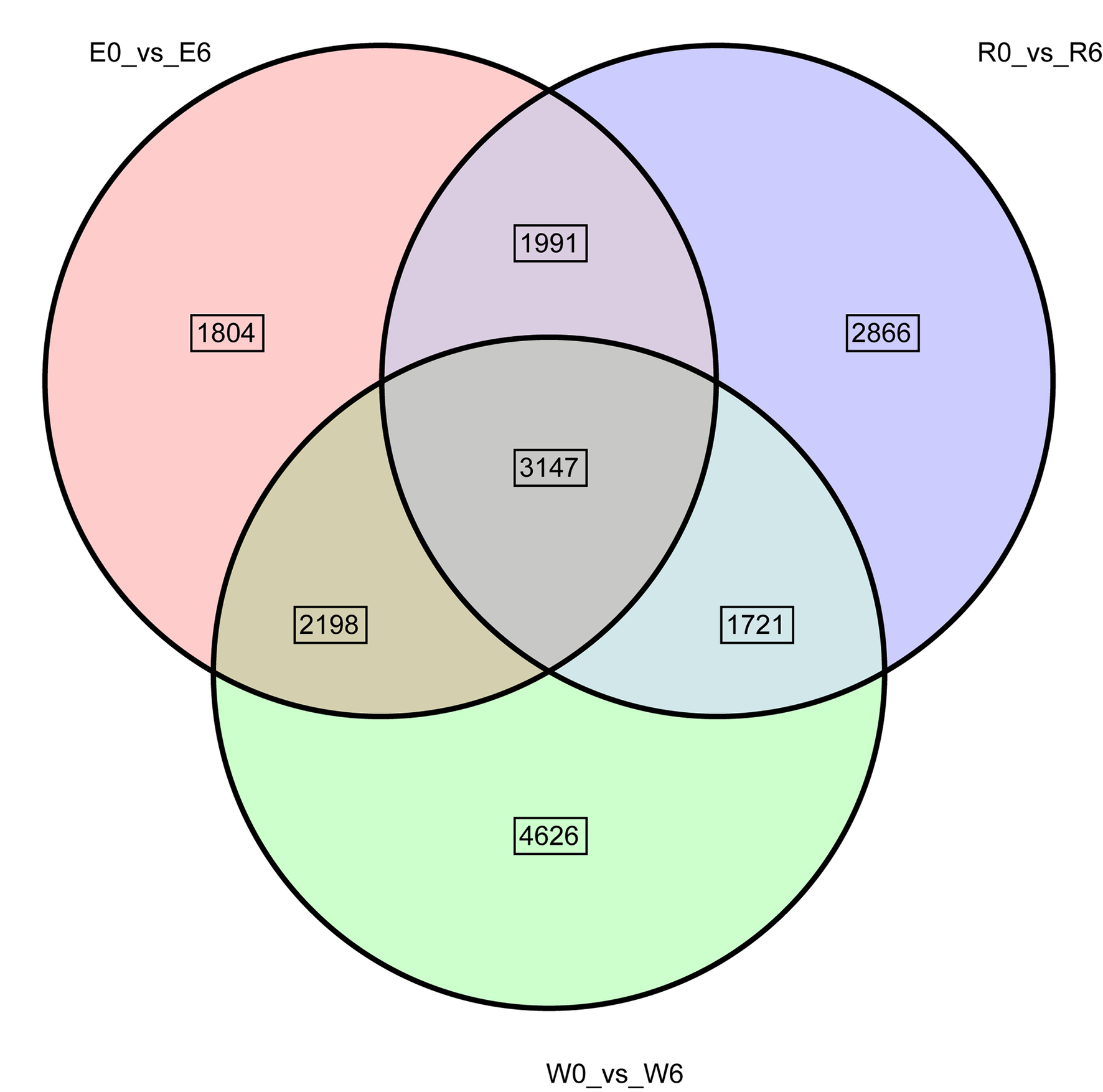

Supplement: Supplementary Figure 5 — Venn diagrams of differentially expressed genes in WT and transgenic wheat lines before and after drought stress. W0, wild type drought stress 0 h; W6, wild type drought stress 6 h; E0, TaNRX1-OE-3 drought stress 0 h; E6, TaNRX1-OE-3 drought stress 6 h; R0, TaNRX1-RNAi-6 drought stress 0 h; R6, TaNRX1-RNAi-6 drought stress 6 h. [file Image_5.TIF]

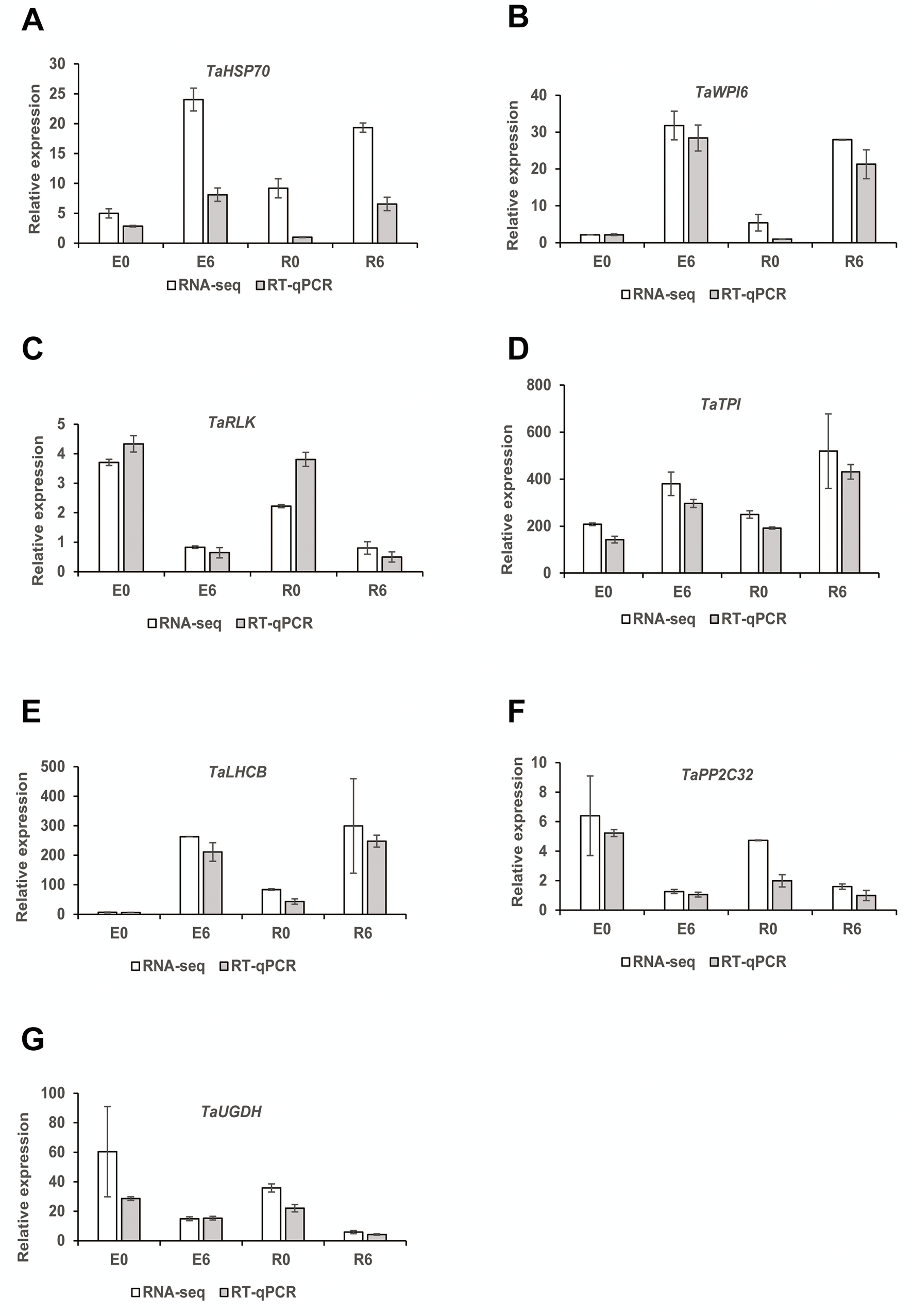

Supplement: Supplementary Figure 6 — RT-qPCR verification of RNA-seq results. (A) Relative expression of 70 kDa heat shock protein. (B) Relative expression of WPI6 gene. (C) Relative expression of receptor-like protein kinase. (D) Relative expression of triosephosphate isomerase. (E) Relative expression of chloroplast a/b binding protein. (F) Relative expression of protein phosphatase 2C. (G) Relative expression of UDP-glucose-6-dehydrogenase. Data represent the mean ± SD (n = 3). E0, TaNRX1-OE-3 drought stress 0 h; E6, TaNRX1-OE-3 drought stress 6 h; R0, TaNRX1-RNAi-6 drought stress 0 h; R6, TaNRX1-RNAi-6 drought stress 6 h. The 2–ΔΔ CT method was used to calculate the relative expression levels of genes. [file Image_6.TIF]
